# Multiple Cross Displacement Amplification Coupled With Gold Nanoparticles-Based Lateral Flow Biosensor for Detection of the Mobilized Colistin Resistance Gene *mcr-1*

**DOI:** 10.3389/fcimb.2019.00226

**Published:** 2019-06-27

**Authors:** Lin Gong, Ernan Liu, Jie Che, Juan Li, Xiaoli Liu, Huiqiong Xu, Jiansheng Liang

**Affiliations:** ^1^Department of Disinfection and Pest Control, Wuhan Centers for Disease Prevention and Control, Wuhan, China; ^2^State Key Laboratory for Infectious Disease Prevention and Control, Collaborative Innovation Center for Diagnosis and Treatment of Infectious Disease, Chinese Center for Disease Control and Prevention, National Institute for Communicable Disease Control and Prevention, Beijing, China

**Keywords:** *mcr-1*, colistin resistance, multiple cross displacement amplification, lateral flow biosensor, detection assay

## Abstract

Fast dissemination of the mobilized colistin resistance (*mcr*) gene *mcr-1* in *Enterobacteriaceae* causes a huge threat to the treatment of severe infection. In the current report, a multiple cross displacement amplification (MCDA) coupled with the detection of amplified products by gold nanoparticles-based lateral flow biosensor (LFB) assay (MCDA-LFB) was established to identify the *mcr-1* gene with simpleness, rapidity, specificity, and sensitivity. The MCDA-LFB assay was performed at a isothermal temperature (63°C) for only 30 min during the amplification stage, and the reaction products were directly identified by using LFB which obtained the result within 2 min. The entire process of experiments, from templates extraction to result judging, was accomplished in <60 min. For the analytical specificity of this method, all of the 16 *mcr-1*-producing strains were positive, and all of the non-*mcr-1* isolates produced the negative results. The sensitivity of *mcr-1*-MCDA-LFB assay was as little as 600 fg of plasmid DNA per reaction in pure culture, and approximately 4.5 × 10^3^ CFU/mL (~4.5 CFU/reaction) in spiked fecal samples. Therefore, this technique established in the present study is suitable for the surveillance of *mcr-1* gene in clinic and livestock industry.

## Introduction

The rapid increase of carbapenem-resistant *Enterobacteriaceae* (CRE) expressing *Klebsiella pneumoniae* carbapenemase (KPC), New Delhi metallo-blactamase (NDM) and oxacillinase (OXA) OXA-48 has risen serious concerns in clinic. Colistin, a “last resort” antibiotic, has a crucial role for treating the infection caused by those species (Nation and Li, 2009). Therefore, the number of colistin consumptions increasing along with the global augment of CRE will bring about the risk of emerging resistance (Gelband et al., 2015).

Resistance to colistin was linked with chromosomal resistance mechanisms in varieties of strains in the past (Olaitan et al., 2014). Since a new mobilized colistin resistance gene, *mcr-1*, carried by plasmid in an *Escherichia coli* was first reported in China in 2015 (Liu et al., 2016), which has been identified in numerous countries. China, Germany and Vietnam carry an important proportion of positive samples (Wang et al., 2018a). The *mcr-1*-positive bacterial species include *Salmonella enterica, E. coli, Escherichia fergusonii, Enterobacter aerogenes, K. pneumoniae, Citrobacter braaki*, and *Klebsiella aerogenes* (Doumith et al., 2016; Li et al., 2016; Stoesser et al., 2016; Zeng et al., 2016; Sennati et al., 2017; Wang et al., 2017a, 2018a). Besides discovered in Clinical samples, *mcr-1* is also detected from environmental settings: meat and vegetable products purchased from markets, Animal feces collected from farms, fecal samples of pets gathered from pet hospital, river water, and seawater (Chen et al., 2017). The wide dissemination of *mcr-1* across diversified species is benefited from many types of *mcr-1*-bearing plasmids covering IncHI2, IncI2, IncFI, IncX4, and IncX1-X2 hybrid type (McGann et al., 2016; Sun et al., 2016, 2017; Yang et al., 2016; Guo et al., 2017). Similarly, the genetic environments of *mcr-1* gene also impact its transmission. A global data set of roughly 500 isolates producing *mcr-1* analyzed by whole-genome sequencing (WGS) has revealed that an initial mobilized event of *mcr-1* is mediated by an ISApl1-*mcr-1-orf*-ISApl1 transposon around 2006 (Wang et al., 2018a). The horizontal transfer of *mcr-1* gene causing inflation of colistin-resistant isolates will lead to the shortage of effective measures for treating infections with multidrug-resistant bacteria. Therefore, a rapid, sensitive and specific diagnostic assay for *mcr-1* detection is imperative to devise.

Currently, several categories of molecular diagnostic methodologies including conventional polymerase chain reaction (PCR) and real-time PCR methods have been devised to identify *mcr-1* gene (Bontron et al., 2016). Nevertheless, the requirements of highly sophisticated devices, strictly experimental environments and well-trained personnel restrict those techniques to apply in resource-challenged areas and “on-site” detection (Niu et al., 2018). Recently, multiple cross displacement amplification (MCDA), a novel nucleic acid amplification technique, has been utilized in detection of bacterial agents, such as *Listeria monocytogenes* and methicillin-resistant *Staphylococcus aureus* (MRSA) (Wang et al., 2017b, 2018c). The approach was based on isothermal strand-displacement polymerization reaction, five pairs of primers were designed to amplify the corresponding regions of target sequence. Without the denaturing step, the primers of high concentration could bind to the template at a isothermal temperature. Several single stem-loop DNA structures and single-stranded DNAs were yielded with the primers binding and the reaction chain extending. Then, those DNA products were exponentially amplified as the reaction progressed (Wang et al., 2015). With the advantages of rapidity, specificity and sensitivity, MCDA operated in a simple heater can yield amplifcons from a few colonies (Wang et al., 2017b, 2018b), the amplicons are identified by a gold nanoparticles-based lateral flow biosensor (LFB) subsequently.

In this study, a MCDA-LFB assay for the rapid detection of *mcr-1* was established, and the sensitivity and specificity of above method in pure culture and in spiked fecal samples were analyzed.

## Materials and Methods

### Reagents and Instruments

Bacterial genomes extraction kits were obtained from Beijing ComWin Biotech Co., Ltd. (Beijing, China). QIAGEN plasmid kits and QIAamp fast DNA stool mini kits were purchased from Qiagen Co., Ltd. (Beijing, China). Isothermal amplification kits (including reaction buffer and Bst DNA polymerase 2.0), colorimetric indicator (Malachite Green), and disposable LFB were provided by BeiJing-HaiTaiZhengYuan Technology Co., Ltd. (Beijing, China). The heating thermostat (MTH-100) was purchased from Hangzhou MiU Instruments Co., Ltd. (Hangzhou, China). The UV transilluminator (UVsolo touch) was obtained from Analytik Jena (Jena, Germany). Nanodrop instrument (ND-2000) was purchased from Thermo Fisher Scientific Co., Ltd. (Massachusetts, America).

### Bacterial Isolates and Genomic Template Preparation

A total of 59 organisms consisting of 16 *mcr-1*-postive isolates and 43 non-*mcr-1* bacteria were used in this study (Table 1). The confirmation of all genes used conventional PCR and sequencing. The *mcr-1*-postive bacterial species included 5 *E. fergusonii*, 10 *E. coli*, and 1 *Salmonella enteritidis*. The mcr-1-negative isolates involved KPC-2-postive stains (*K. pneumoniae* and *Pseudomonas aeruginosa*), NDM-1-postive isolates (*E. coli, Enterobacter cloacae*, and *K. pneumoniae*), NDM-5-producing *E. coli*, IMP-4-producing *E. coli*, and *mcr-1/*carbapenemase-negative species (*Acinetobacter baumannii, P. aeruginosa, Serratia marcescens, E. coli, S. aureus, Enterococcus faecalis, and Enterococcus faecium*). According to the handling instruction, the genomic DNA of all strains was extracted by bacterial genomes extraction kits, the plasmid DNA of *mcr-1-*producing *E. fergusonii* (ICDC-ZG2016M34-3) acted as a representative sample for optimization of reaction condition and sensitivity detection was acquired by QIAGEN Plasmid Kits, and quantified by a Nanodrop ND-2000 instrument.

**Table 1:** Strains used in this study.

**Genotype^a^**	**Bacteria species**	**The source of strains^b^**	**No. of isolates**
*mcr-1*	*Escherichia fergusonii*	ICDC (ZG2016M34-3)	1
	*Escherichia fergusonii*	ICDC	3
	*Escherichia fergusonii*	WHCDC	1
	*Escherichia coli*	ICDC	8
	*Escherichia coli*	WHCDC	2
	*Salmonella enteritidis*	ICDC	1
KPC-2	*Klebsiella pneumoniae*	WHCDC	10
	*Pseudomonas aeruginosa*	WHCDC	1
NDM-1	*Escherichia coli*	WHCDC	4
	*Enterobacter cloacae*	WHCDC	2
	*Klebsiella pneumoniae*	WHCDC	4
NDM-5	*Escherichia coli*	WHCDC	2
IMP-4	*Escherichia coli*	WHCDC	2
NO	*Acinetobacter baumannii*	WHCDC	3
	*Pseudomonas aeruginosa*	WHCDC	3
	*Serratia marcescens*	WHCDC	2
	*Escherichia coli*	WHCDC	2
	*Staphylococcus aureus*	WHCDC	4
	*Enterococcus faecalis*	WHCDC	2
	*Enterococcus faecium*	WHCDC	2

a NO, the stains did not carry above genes.

b *ICDC, National Institute for Communicable Disease Control and Prevention, Chinese Center for Disease Control and Prevention. WHCDC, Wuhan Centers for Disease Prevention and Control*.

### Primers Design of MCDA Assay

**Figure 1:**
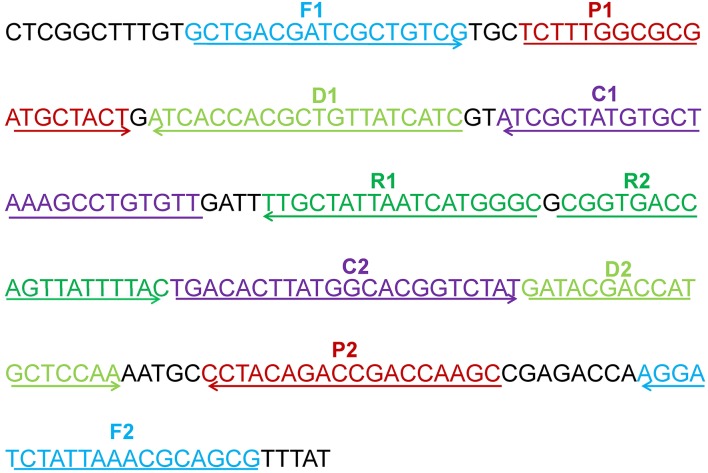
The location of the five primers pairs on the partial *mcr-1* gene sequence. Different pairs of primers were represented by various of colors. The arrowed lines showed the amplification direction of primers.

A set of five pairs of primers, including 2 displacement primers (F1 and F2), 2 cross primers (CP1 and CP2), and 3 pairs of amplification primers (C1, C2, D1, D2, R1, and R2), targeted 10 distinct regions more than 150 bp on *mcr-1* gene (Wang et al., 2015). Two softwares named Primer Premier 6.0 and PrimerExplorer V4 were used to design the 10 MCDA primers based on *mcr-1* gene (GenBank accession number: KX886345). The dimer and hairpin structures of all primers were detected by Integrated DNA Technologies design tools (Wang et al., 2016), and the specificity of which was analyzed by using Basic Local Alignment Search Tool (Blast). The relevant information of primers pairs regarding positions and sequences was displayed in Figure 1 and Table 2. Furthermore, The FITC (Fluorescein isothiocyanate) and biotin labeled at 5′end of the C1 and D1 primers, respectively, and the labeled primers were named as C1^*^ and D1^*^. All of the primers were synthesized and purified by Sangon Biotechnology Co., Ltd. (Shanghai, China) at HPLC purification grade.

**Table 2:** Primers of the MCDA assay to identify the *mcr-1* gene.

**Primers^a^**	**Sequences and modifications (5^**′**^-3^**′**^)**	**Length^b^**
F1	GCTGACGATCGCTGTCG	17 nt
F2	CGCTGCGTTTAATAGATCCT	20 nt
CP1	AACACAGGCTTTAGCACATAGCGATCTTTGGCGCGATGCTACT	43 mer
CP2	TGACACTTATGGCACGGTCTATGCTTGGTCGGTCTGTAGG	40 mer
C1^*^	FITC-AACACAGGCTTTAGCACATAGCGAT	25 nt
C2	TGACACTTATGGCACGGTCTAT	22 nt
D1^*^	biotin-GATGATAACAGCGTGGTGAT	20 nt
D2	GATACGACCATGCTCCAA	18 nt
R1	GCCCATGATTAATAGCAA	18 nt
R2	CGGTGACCAGTTATTTTAC	19 nt

a C1^*^, 5**′** end of C1 was labeled with FITC; D1^*^, 5**′** end of D1 was labeled with biotin

b *nt meant the nucleotide; mer referred to monomeric*.

### The Standard MCDA Assay

The MCDA reaction systems were performed according to the previous studies (Wang et al., 2016). Each reaction, the total volumes of 25 μL, included reaction buffer (12.5 μL), Bst DNA polymerase 2.0 (1 μL), colorimetric indicator (1 μL), cross primers (1.6 μM each), displacement primers (0.4 μM each), amplification primers (0.4 μM each), and 1 μL of DNA template. The NDM-1-positive *E. coli* (WHCDC-WH67) and KPC-2-producing *K. pneumoniae* (WHCDC-WH108) were regarded as the negative controls, and the distilled water was served as the blank control. To assess the optimal reaction temperature of *mcr-1*-MCDA, the MCDA amplification systems were executed with a constant temperature in the range of 60–67°C for 40 min.

The MCDA reaction products were analyzed by three detection methods including 2% agarose gel electrophoresis, colorimetric indicator, and LFB (Wang et al., 2017b). When employing gel electrophoresis, 3 μL of reaction mixtures were run at 110 volts for 60 min. A ladder of multiple bands could be observed in the positive reactions, but not in the negative and blank controls. Reaction products were detected by using colorimetric indicator, the color of amplified products remained unchanged. Nevertheless, the negative and blank controls reactions changed from blue to colorless. The material, theory and operation procedure of LFB were previously described by Wang et al. (2017b). 0.2 μL of amplicons followed by three drops of the running buffer consisting of 1% Tween 20 and 0.01 mol/L phosphate-buffered saline were added to the well of sample pad (Niu et al., 2018). After 1–2 min, two red lines named test line (TL) and control line (CL), respectively, could be visualized in positive products, while only the CL was observed for the negative and blank control.

### Sensitivity and Specificity of the *mcr-1-*MCDA-LFB Assay

The plasmid DNA of *E. fergusonii* ICDC-ZG2016M34-3 was serially diluted (6 ng, 60 pg, 600 fg, 60 fg, and 6 fg per μl) for sensitivity analysis of *mcr-1*-MCDA-LFB detection. The colorimetric indicator and agarose gel electrophoresis were carried out simultaneously. Each test was repeated three times. The specificity of *mcr-1*-MCDA-LFB assay was evaluated with the DNA templates of 16 *mcr-1-*producing strains and 43 non-*mcr-1* strains (Table 1). The specificity evaluations were confirmed twice.

### The Optimal Amplification Time

In order to screen the optimal time for the *mcr-1*-MCDA-LFB assay, The MCDA mixture was completed at the reaction temperature in the range from 10 to 40 min at 10 min intervals. Subsequently, the MCDA products were detected by LFB detection. Each amplification time was operated at two times.

### *mcr-1*-MCDA-LFB Detection in Spiked Fecal Samples

The fecal samples were obtained from a healthy man in Wuhan, China. *Mcr-1* gene was not detected in those samples according to the microbial culture and PCR identification. The volumes of 100 μL were taken out from the *mcr-1*-positive *E. fergusonii* ICDC-ZG2016M34-3 cultures when the optical density (OD) of that reached to 0.6. The suspensions were serially diluted (10^−1^−10^−8^), and the aliquots of 100 μL dilutions (10^−3^−10^−6^) were incubated on nutrient agar plates with three replicates, colony forming units (CFUs) were counted subsequently. One hundred microliter of diluted *mcr-1*-producing cultures (10^−2^−10^−7^) with known amounts (4.5 × 10^6^−4.5 × 10^1^ CFU/ mL) was, respectively, added to 0.2 g of fecal sample and mixed well. DNA templates were extracted with the manufacturer's protocol by using QIAamp fast DNA stool mini kits. The extracted genomic DNA was dissolved in 100 μl of elution buffer, and 1 μl of which was used for MCDA-LFB detection as templates. A non-spiked feces sample was tested as negative control. The products of MCDA were also detected by colorimetric indicator and 2% agarose gel electrophoresis. The evaluation assay for limit of detection in fecal samples was conducted triplicate.

### Conventional PCR

*Mcr*-1 gene was amplified by conventional PCR, primers: *mcr*-1-F, 5′-CGG TCA GTC CGT TTG TTC-3′; *mcr-*1-R, 5′-CGG TCA GTC CGT TTG TTC-3′. And amplicons were subsequently sequenced (Liu et al., 2016). The LOD of *mcr-1*-MCDA-LFB and conventional PCR in pure culture and in fecal samples were compared, respectively.

## Results

### The Verification of *mcr-1* MCDA Products

**Figure 2:**
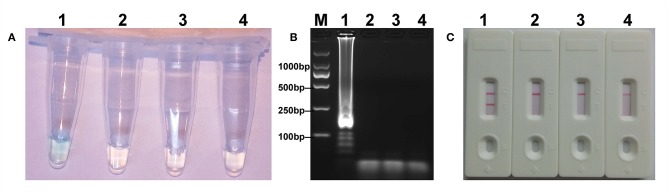
Confirmation of the *mcr-1*-MCDA-LFB assay. There were three detection methods to identify MCDA products: **(A)** colorimetric indicators, **(B)** agarose gel electrophoresis, **(C)** LFB. Four samples were tested: (1) *mcr-1*-positive strain of *Escherichia fergusonii* (ICDC-ZG2016M34-3); (2) NDM-1-positive *Escherichia coli* (WHCDC-WH67); (3) KPC-2-producing *Klebsiella pneumoniae* (WHCDC-WH108); (4) distilled water. Only the amplification with *Escherichia fergusonii* (ICDC-ZG2016M34-3) showed the positive.

The *mcr-1* MCDA assays were performed at a constant temperature (63°C) for 40 min to verify the availability of MCDA primers. Positive reaction appeared with DNA from *mcr-1*-producing *E. fergusonii* (ICDC-ZG2016M34-3), but not with NDM-1-positive *E. coli* (WHCDC-WH67), KPC-2-producing *K. pneumoniae* (WHCDC-WH108), and the blank control (Figure 2). Therefore, the primers of *mcr-1*-MCDA was suitable for establishment of the MCDA-LFB assay to detect *mcr-1* gene.

### Temperature Optimization for *mcr-1*-MCDA-LFB Assay

**Figure 3:**
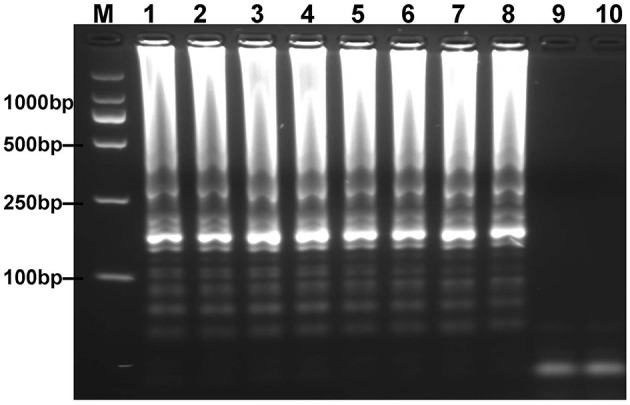
Optimal reaction temperature for *mcr-1*-MCDA assay was determined by using agarose gel electrophoresis. The plasmid DNA of *Escherichia fergusonii* (ICDC-ZG2016M34-3) (6 pg) was amplified in different temperatures. Lane 1–8 represented the amplification temperature in the range from 60 to 67°C (1°C intervals); lane 9, negative control (6 pg of *Escherichia coli* WHCDC-WH67 genomic DNA); lane 10, blank control (DW).

To optimize the reaction temperature of MCDA-LFB assay during the amplification stage, the plasmid DNA of *E. fergusonii* (ICDC-ZG2016M34-3) at the level of 6 pg per reaction was used as the templates. A series of temperatures (ranging from 60 to 67°C, with 1°C intervals) was compared for amplifying efficiency of MCDA-LFB assay by employing 2% agarose gel electrophoresis. The result showed that 62 and 63°C were the better candidates for this method (Figure 3). Therefore, the reaction temperature of 63°C was performed for the subsequent MCDA-LFB experiments.

### Sensitivity and Specificity of MCDA-LFB Assay for *mcr-1*

**Figure 4:**
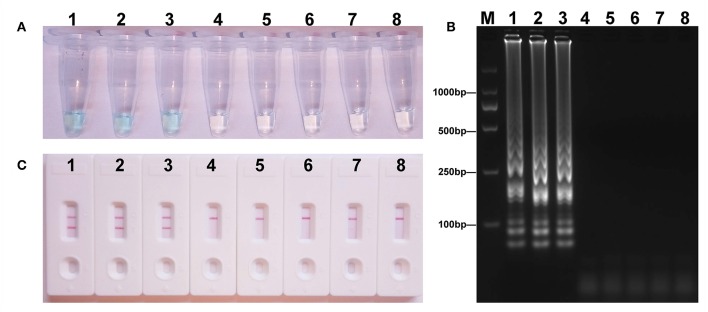
Sensitivity of the *mcr-1*-MCDA assay using serially diluted plasmid DNA with *Escherichia fergusonii* ICDC-ZG2016M34-3. Three detection techniques, including colorimetric indicators **(A)**, gel electrophoresis **(B)**, and LFB **(C)**, were applied for testing MCDA products. Tubes **(A)**/lanes **(B)**/biosensors **(C)** 1–8 represented the plasmid DNA levels of 6 ng, 60 pg, 600 fg, 60 fg, and 6 fg per reaction, negative control (NDM-1-positive *Escherichia coli* WHCDC-WH67 genomic DNA, 6 pg per reaction), negative control (KPC-2-producing *Klebsiella pneumoniae* WHCDC-WH108 genomic DNA, 6 pg per reaction), and blank control (DW).

To acquire the detection limit of this assay, serial dilutions of *E. fergusonii* ICDC-ZG2016M34-3 plasmid DNA were used in *mcr-1*-MCDA-LFB assay. It indicated that the threshold was as little as 600 fg of plasmid DNA (Figure 4). The same results were observed by using colorimetric indicator and agarose gel electrophoresis analysis.

**Figure 5:**
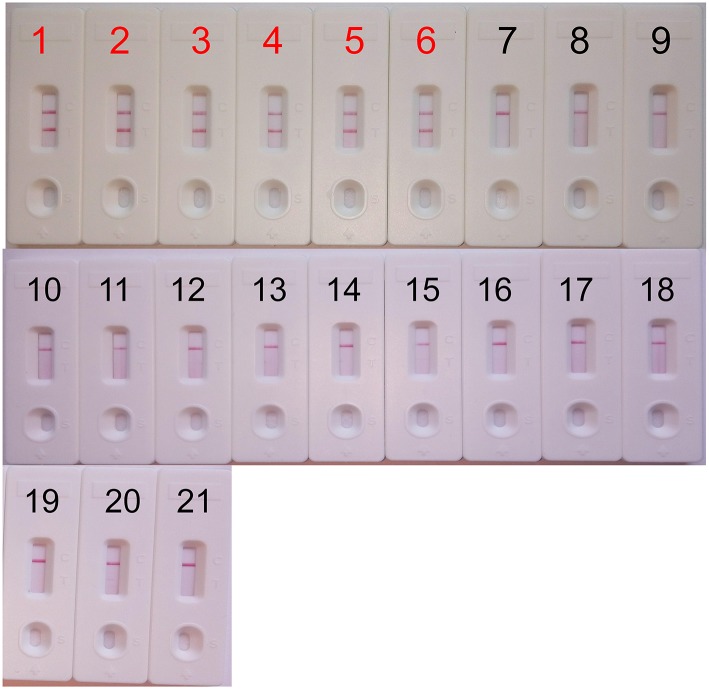
Specificity of the *mcr-1*-MCDA-LFB assay. Biosensor 1, *mcr-1*-positive *Escherichia fergusonii* ICDC-ZG2016M34-3. Biosensor 2, *mcr-1*-positive *Escherichia fergusonii* from ICDC. Biosensor 3, *mcr-1*-positive *Escherichia fergusonii* from WHCDC. Biosensor 4, *mcr-1*-producing *Escherichia coli* from ICDC. Biosensor 5, *mcr-1*-producing *Escherichia coli* from WHCDC. Biosensor 6, *mcr-1*-producing *Salmonella enteritidis* from ICDC. Biosensors 7–17, KPC-2-postive *Klebsiella pneumoniae*, KPC-2-postive *Pseudomonas aeruginosa*, NDM-1-postive *Escherichia coli*, NDM-1-postive *Enterobacter cloacae*, NDM-1-postive *Klebsiella pneumoniae*, NDM-5-postive *Escherichia coli*, IMP-4-postive *Escherichia coli, Acinetobacter baumannii* carring non*-mcr-1/*non*-*carbapenemase gene, *Pseudomonas aeruginosa* carring non*-mcr-1/*non carbapenemase gene, *mcr-1/*carbapenemase-negative *Serratia marcescens, mcr-1/*carbapenemase-negative *Escherichia coli*, all of the stains derived from WHCDC. Biosensor 18, blank control (DW). Biosensors 19–21, *Staphylococcus aureus* carring non*-mcr-1/*non*-*carbapenemase gene, *Enterococcus faecalis* carring non*-mcr-1/*non carbapenemase gene, *mcr-1/*carbapenemase-negative *Enterococcus faecium*, those stains were isolated from WHCDC.

The analytical specificity of the *mcr-1-*MCDA-LFB assay was assessed with genomic DNA extracted from 16 *mcr-1*-producing strains and 43 non-*mcr-1* isolates. As shown in Figure 5, all products derived from the strains carrying *mcr-1* gene exhibited two red bands (TL and CL) in the LFB, but each sample from the *mcr-1-*negative organisms and blank control yielded only one red band. The results certified that the MCDA-LFB assay had a complete specificity for *mcr-1* detection.

### Optimization of the Time for *mcr-1*-MCDA-LFB Assay

**Figure 6:**
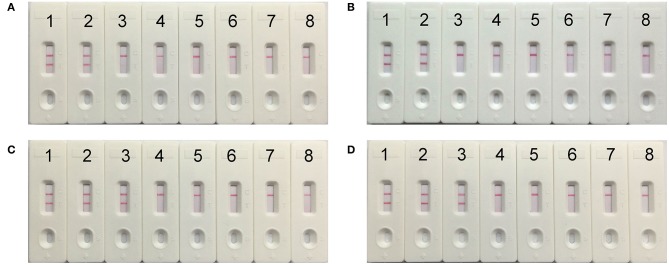
The optimal reaction time for *mcr-1*-MCDA-LFB assay. Four different amplification times, covering 10 min **(A)**, 20 min **(B)**, 30 min **(C)**, and 40 min **(D)**, were compared at 63°C. Biosensors 1–8 represented the plasmid DNA levels of 60 pg, 6 pg, 600 fg, 60 fg, and 6 fg of *Escherichia fergusonii* ICDC-ZG2016M34-3, negative control (6 pg of NDM-1-positive *Escherichia coli* WHCDC-WH67 genomic DNA), negative control (6 pg of KPC-2-producing *Klebsiella pneumoniae* WHCDC-WH108 genomic DNA) and blank control (DW).

To evaluate the optimum time, four reaction times (10–40 min at 10 min intervals) were tested for the *mcr-1*-MCDA-LFB assay during the amplification stage. The *mcr-1*-producing *E. fergusonii* (ICDC-ZG2016M34-3) plasmid DNA, 600 fg/μl (the LOD of the method), did not gain the positive results until the reaction had operated for 30 min (Figure 6). Hence, the amplification time of 30 min at 63°C was considered as an optimal reaction condition for the current assay.

### Application of MCDA-LFB to *mcr-1*-spiked Fecal Samples

**Figure 7:**
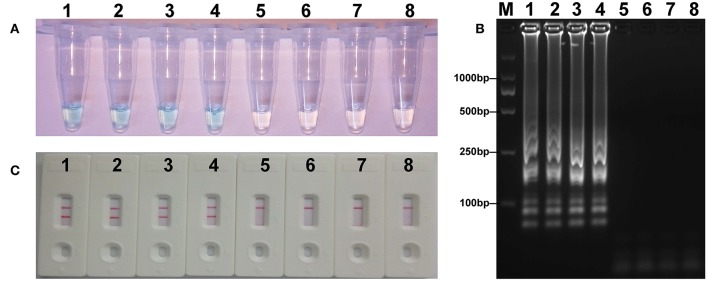
Sensitivity of *mcr-1-*MCDA-LFB assay in spiked feces samples. Three measurement techniques, including colorimetric indicators **(A)**, gel electrophoresis **(B)**, and LFB **(C)**, were applied for testing MCDA products. Tubes **(A)**/lanes **(B)**/biosensors **(C)** 1–8 represented the DNA levels of 4.5 × 10^3^, 4.5 × 10^2^, 4.5 × 10^1^, 4.5 × 10^0^, 4.5 × 10^−1^, and 4.5 × 10^−2^ CFU per reaction, negative control (non-spiked feces sample) and blank control (DW).

The LOD for strains expressing *mcr-1* in fecal samples was determined to assess the practical application of the established assay. The detected threshold of *mcr-1*-positive bacteria was approximately 4.5 × 10^3^ CFU/mL (~4.5 CFU/reaction) in 0.2 g fecal samples spiked with 100 μl of dilutions of strains (Figure 7). The results of other subjects including the lower suspensions concentrations, negative control, and blank control were negative. As the same to the aforementioned experiments, detection of the amplicons with three methods got an equivalent conclusion.

### The Detection Limit of Conventional PCR

The detection limit of the conventional PCR assay was 6 pg of plasmid DNA per microliter in pure culture and 4.5 × 10^4^ CFU/mL (~45 CFU/reaction) in spiked fecal samples. *Mcr-1*-MCDA-LFB method was thus highly sensitive, 10-fold more so than that of PCR (Table 3).

**Table 3:** Detection limit of the *mcr-1*-MCDA-LFB assay and conventional PCR.

	**Detection limit**	
	**Conventional PCR**	***Mcr-1*-MCDA-LFB assay**
Purified plasmid DNA	6 pg/uL	600 fg/uL
DNA-spiked specimens	~45 CFU/reaction	~4.5 CFU/reaction

## Discussion

Polymyxins (polymyxin B and colistin) have nearly become last-resort drugs for treating the severe infections caused by multidrug-resistant or pan-resistant *Enterobacteriaceae* (Wang et al., 2017a). The defensive line will be destroyed by the emergence of *mcr-1*-positive strains resisting to colistin. Moreover, the *mcr-1* gene mediated by plasmids or transposons can transfer in different species freely. Thus, the isolates carrying *mcr-1* gene will undoubtedly become a major issue for public health. Under the circumstances, a convenient and fast technique for detection of *mcr-1* in various samples is of great importance. Here, an approach was reported to detect target gene by MCDA united with lateral flow biosensor (MCDA-LFB). In the MCDA-LFB assay, the high specificity was guaranteed, as a set of 10 primers was employed for specially amplifying the target sequence. The specificity of *mcr-1*-MCDA-LFB was successfully confirmed with the genomic templates extracted from *mcr-1*-producing strains and non-*mcr-1* organisms, and the results were positive for all *mcr-1*-positive isolates, but negative for non-*mcr-1* isolates and blank control. Therefore, the diagnostic test based on MCDA-LFB for the detection of *mcr-1* in bacteria identifies target gene with high selectivity.

The MCDA products can be analyzed with LFB, colorimetric indicator and agarose gel electrophoresis, respectively. LFB, as a detection technique by observing the number of red lines on sensor bar, is more objective than colorimetric indicator, which reports the result through color change. Maybe the latter is in trouble when the concentration of target gene is very low (Wang et al., 2017c). Likewise, LFB is more rapid and convenient than gel electrophoresis, which requires the use of an additional operation procedure and complex equipment. Hence, LFB will be a better candidate for the results' judge of MCDA products.

Besides specificity, the superb sensitivity is also very important for the newly established assay. The *mcr-1*-MCDA-LFB method sufficed to detect as little as 600 fg of *mcr-1*-positive plasmid DNA per microliter in pure culture and 4.5 × 10^3^ CFU/mL (~4.5 CFU/reaction) in fecal samples spiked with 100 μl of strains. This technique has the same sensitivity to *mcr-1*-LAMP described in previously report (Zou et al., 2017), and is 10 times more sensitive than that of conventional PCR. Moreover, in spite of the equivalent specificity and sensitivity in both isothermal amplification assays, the *mcr-1*-MCDA-LFB method which expends less reaction time than the other will provide faster detection for *mcr-1*. Likewise, although the Real-time PCR and MALDI-TOF MS-based method could also test the *mcr-1* gene in low limit of detection and less time, respectively (Bontron et al., 2016; Dortet et al., 2018), they needed expensive apparatus and immaculately experimental condition that were not well-equipped in resource-challenged fields, especially in the livestock industry where a large quantity of stains carrying *mcr* gene were identified (Dortet et al., 2018).

The *mcr-1*-MCDA-LFB assay only required a constant reaction temperature at 63°C. The entire process of experiments, including sample processing (25 min), isothermal amplification (30 min), and detection (2 min), could be accomplished in <60 min. Herein, this assay economizes the test time and device, and is suitable for timely identification on the spot particularly.

In conclusion, we devised a reliable MCDA-LFB assay for the detection of *mcr-1* with simplicity, rapidity, and low-cost facility. The LOD of this assay was only 600 fg per reaction with pure culture, and the specificity was 100% according to the trial results. From the above, the *mcr-1*-MCDA-LFB assay built in this study will greatly improve the detection efficiency for the monitor of target gene in practical application.

## Data Availability

The raw data supporting the conclusions of this manuscript will be made available by the authors, without undue reservation, to any qualified researcher.

## Author Contributions

LG and JiL designed the experiments. LG and EL carried out the experiments. JuL and JC contributed the partial isolates. XL and HX provided the materials and reagents. LG analyzed the related data and wrote the paper.

### Conflict of Interest Statement

The authors declare that the research was conducted in the absence of any commercial or financial relationships that could be construed as a potential conflict of interest. The reviewer LD declared a shared affiliation, with no collaboration, with several of the authors, JC and JuL, to the handling editor at time of review.

## References

[B1] Bontron S. Poirel L. Nordmann P. (2016). Real-time PCR for detection of plasmid-mediated polymyxin resistance (*mcr-1*) from cultured bacteria and stools. J. Antimicrob. Chemother. 71, 2318–2320. 10.1093/jac/dkw139 27121402

[B2] Chen K. Chan E. W. Xie M. Ye L. Dong N. Chen S. (2017). Widespread distribution of *mcr-1*-bearing bacteria in the ecosystem, 2015 to 2016. Euro. Surveill. 22:17-00206. 10.2807/1560-7917.ES.2017.22.39.17-00206 29019312 PMC5709956

[B3] Dortet L. Bonnin R. A. Pennisi I. Gauthier L. Jousset A. B. Dabos L.. (2018). Rapid detection and discrimination of chromosome- and MCR-plasmid- mediated resistance to polymyxins by MALDI-TOF MS in *Escherichia coli*: the MALDIxin test. J. Antimicrob. Chemother. 73, 3359–3367. 10.1093/jac/dky330 30184212

[B4] Doumith M. Godbole G. Ashton P. Larkin L. Dallman T. Day M.. (2016). Detection of the plasmid-mediated *mcr-1* gene conferring colistin resistance in human and food isolates of *Salmonella enterica* and *Escherichia coli* in England and Wales. J. Antimicrob. Chemother. 71, 2300–2305. 10.1093/jac/dkw093 27090630

[B5] Gelband H. Miller-Petrie M. K. Pant S. Gandra S. Levinson J. Barter D. (2015). The state of the world's antibiotics 2015. Wound Heal. South. Afr. 8, 30–34. Available online at: https://hdl.handle.net/10520/EJC180082

[B6] Guo Q. Su J. McElheny C. L. Stoesser N. Doi Y. Wang M. (2017). IncX2 and IncX1-X2 hybrid plasmids coexisting in fosA6-producing *Escherichia coli*. Antimicrob. Agents Chemother. 61, e00536–e00517. 10.1128/AAC.00536-17 28438937 PMC5487653

[B7] Li Z. Tan C. Lin J. Feng Y. (2016). Diversified variants of the *mcr-1*-carrying plasmid reservoir in the swine lung microbiota. Sci. China Life Sci. 59, 971–973. 10.1007/s11427-016-5111-9 27520829

[B8] Liu Y. Y. Wang Y. Walsh T. R. Yi L. X. Zhang R. Spencer J.. (2016). Emergence of plasmid-mediated colistin resistance mechanism *mcr-1* in animals and human beings in China: a microbiological and molecular biological study. Lancet Infect. Dis. 16, 161–168. 10.1016/S1473-3099(15)00424-7 26603172

[B9] McGann P. Snesrud E. Maybank R. Corey B. Ong A. C. Clifford R. (2016). *Escherichia coli* harboring *mcr-1* and *bla*_CTX−M_ on a novel IncF plasmid: first report of *mcr-1* in the United States. Antimicrob. Agents Chemother. 60, 4420–4421. 10.1128/AAC.01103-16 27230792 PMC4914657

[B10] Nation R. L. Li J. (2009). Colistin in the 21st century. Curr. Opin. Infect. Dis. 22, 535–543. 10.1097/QCO.0b013e328332e672 19797945 PMC2869076

[B11] Niu L. Zhao F. Chen J. Nong J. Wang C. Wang J.. (2018). Isothermal amplification and rapid detection of *Klebsiella pneumoniae* based on the multiple cross displacement amplification (MCDA) and gold nanoparticle lateral flow biosensor (LFB). PLoS ONE 13:e0204332. 10.1371/journal.pone.0204332 30273362 PMC6166938

[B12] Olaitan A. O. Morand S. Rolain J. M. (2014). Mechanisms of polymyxin resistance: acquired and intrinsic resistance in bacteria. Front. Microbiol. 5:643. 10.3389/fmicb.2014.00643 25505462 PMC4244539

[B13] Sennati S. Di Pilato V. Riccobono E. Di Maggio T. Villagran A. L. Pallecchi L.. (2017). *Citrobacter braakii* carrying plasmid-borne *mcr-1* colistin resistance gene from ready-to-eat food from a market in the Chaco region of Bolivia. J. Antimicrob. Chemother. 72, 2127–2129. 10.1093/jac/dkx078 28333311

[B14] Stoesser N. Mathers A. J. Moore C. E. Day N. P. Crook D. W. (2016). Colistin resistance gene *mcr-1* and pHNSHP45 plasmid in human isolates of *Escherichia coli* and *Klebsiella pneumoniae*. Lancet Infect. Dis. 16, 285–286. 10.1016/S1473-3099(16)00010-4 26774239

[B15] Sun J. Fang L. X. Wu Z. Deng H. Yang R. S. Li X. P.. (2017). Genetic analysis of the IncX4 plasmids: implications for a unique pattern in the *mcr-1* acquisition. Sci. Rep. 7:424. 10.1038/s41598-017-00095-x 28336940 PMC5428312

[B16] Sun J. Li X. Yang R. Fang L. Huo W. Li S.. (2016). Complete nucleotide sequence of an IncI2 plasmid coharboring *bla*_CTX−M−55_ and *mcr-1*. Antimicrob. Agents Chemother. 60, 5014–5017. 10.1128/AAC.00774-16 27216063 PMC4958226

[B17] Wang Q. Sun J. Li J. Ding Y. Li X. P. Lin J.. (2017a). Expanding landscapes of the diversified *mcr-1*-bearing plasmid reservoirs. Microbiome 5:70. 10.1186/s40168-017-0288-0 28683827 PMC5500976

[B18] Wang R. Dorp L. V. Shaw L. P. Bradley P. Wang Q. Wang X.. (2018a). The global distribution and spread of the mobilized colistin resistance gene mcr-1. Nat. Commun. 9:1179. 10.1038/s41467-018-03205-z 29563494 PMC5862964

[B19] Wang Y. Li H. Li D. Li K. Wang Y. Xu J.. (2016). Multiple cross displacement amplification combined with gold nanoparticle-based lateral flow biosensor for detection of *Vibrio parahaemolyticus*. Front. Microbiol. 7:2047. 10.3389/fmicb.2016.02047 28066368 PMC5177632

[B20] Wang Y. Li H. Wang Y. Li H. Luo L. Xu J.. (2017b). Development of multiple cross displacement amplification label-based gold nanoparticles lateral flow biosensor for detection of *Listeria monocytogenes*. Int. J. Nanomed. 12, 473–486. 10.2147/IJN.S123625 28138243 PMC5238772

[B21] Wang Y. Li H. Wang Y. Xu H. Xu J. Ye C. (2018b). Antarctic thermolabile uracil-DNA- glycosylase-supplemented multiple cross displacement amplification using a label-based nanoparticle lateral flow biosensor for the simultaneous detection of nucleic acid sequences and elimination of carryover contamination. Nano. Res. 11, 2632–2647. 10.1007/s12274-017-1893-z

[B22] Wang Y. Li H. Wang Y. Zhang L. Zhang J. Xu J. (2017c). Nanoparticle-based lateral flow biosensor combined with multiple cross displacement amplification for rapid, visual and sensitive detection of *Vibrio cholerae*. FEMS Microbiol. Lett. 15:364 10.1093/femsle/fnx234 29155937

[B23] Wang Y. Wang Y. Ma A. J. Li D. X. Luo L. J. Liu D. X.. (2015). Rapid and sensitive isothermal detection of nucleic-acid sequence by multiple cross displacement amplification. Sci. Rep. 5:11902. 10.1038/srep11902 26154567 PMC4648395

[B24] Wang Y. Yan W. Fu S. Hu S. Wang Y. Xu J.. (2018c). Multiple cross displacement amplification coupled with nanoparticles-based lateral flow biosensor for detection of *Staphylococcus aureus* and identification of methicillin-resistant *S. aureus*. Front. Microbiol. 9:907. 10.3389/fmicb.2018.00907 29867818 PMC5954800

[B25] Yang R. Feng Y. Lv X. Duan J. Chen J. Fang L. (2016). Emergence of NDM-5 and *MCR-1*-producing *Escherichia coli* clone ST648 and ST156 from a single Muscovy duck (*Cairina moschata*). Antimicrob. Agents. Chemother. 60, 6899–6902. 10.1128/AAC.01365-16 27550364 PMC5075103

[B26] Zeng K. J. Doi Y. Patil S. Huang X. Tian G. B. (2016). Emergence of plasmid-mediated *mcr-1* gene in colistin-resistant *Enterobacter aerogenes* and *Enterobacter cloacae*. Antimicrob. Agents. Chemother. 60, 3862–3863. 10.1128/AAC.00345-16 26976876 PMC4879368

[B27] Zou D. Huang S. Lei H. Yang Z. Su Y. He X.. (2017). Sensitive and rapid detection of the plasmid-encoded colistin-resistance gene *mcr-1* in *Enterobacteriaceae* isolates by loop-mediated isothermal amplification. Front. Microbiol. 8:2356. 10.3389/fmicb.2017.02356 29238331 PMC5712548

